# Body-Image Discrepancy and Disordered Eating in Children Aged 10–12: The Roles of Gender, BMI, and Thinness-Related Attributions

**DOI:** 10.3390/nu18060978

**Published:** 2026-03-19

**Authors:** Marios Argyrides, Omer Horovitz, Glykeria Reppa, Kyriaki Kouppa

**Affiliations:** 1 Eating and Appearance Research Laboratory, Neapolis University Pafos, Paphos 8042, Cyprus; g.reppa.1@nup.ac.cy (G.R.); kyriaki.koupa@gmail.com (K.K.); 2 The Physiology and Behavior Laboratory, Tel-Hai Academic College, Qiryat Shemona 1220800, Israel; omerho1@telhai.ac.il; 3 Psychology Department, Tel-Hai Academic College, Qiryat Shemona 1220800, Israel

**Keywords:** body image discrepancy, disordered eating, preadolescents, disordered eating risk, body dissatisfaction, thin-ideal internalization, children, Cyprus

## Abstract

Background/Objectives: Early manifestations of body dissatisfaction and subclinical disordered eating are increasingly recognized as important correlates of later disordered eating, underscoring the need for research in preadolescent populations. This study examined the prevalence of disordered eating risk and tested whether body-figure discrepancy and thinness-related attribution patterns were associated with disordered-eating symptomatology beyond gender and BMI among children aged 10–12 years. Methods: A total of 227 children completed the Children’s Eating Attitudes Test-26 (ChEAT-26), body-figure silhouette measures, and assessments of thinness-related social-emotional, negative, and competence attributions. Results: Overall, 16.3% of participants scored at or above the clinical cutoff for elevated eating-pathology risk, with no significant gender differences. Hierarchical regression analyses showed that the discrepancy between perceived and ideal body figure was significantly associated with disordered eating severity, although the proportion of explained variance was modest. Logistic regression further indicated that each unit increase in body-figure discrepancy was associated with a 37% increase in the likelihood of exceeding the clinical risk threshold. In contrast, thinness-related attribution indices were intercorrelated but were not significantly associated with disordered eating, nor were their effects moderated by gender. Conclusions: These findings indicate that body-image discrepancy is associated with disordered eating in late childhood, at a stage when marked gender differences are not yet evident. Although causal inferences cannot be drawn, the presence of clinically elevated symptom levels and the observed associations highlight the potential importance of early attention to body dissatisfaction in preventive efforts.

## 1. Introduction

The significant impact of subclinical disordered eating behaviors and body dissatisfaction among young individuals emphasizes the critical need for early assessment and intervention strategies [1]. This focus is particularly important given that pervasive body dissatisfaction and disordered eating symptoms are already well established by early adolescence [2]. International research has further demonstrated that body dissatisfaction, dieting behaviors, and weight-related concerns can emerge even before adolescence, with behaviors such as dieting and exercising to lose weight reported in children as young as eight years old [3]. These findings indicate that early psychological correlates of disordered eating are observable across cultural contexts, underscoring the importance of examining such processes during late childhood.

Investigating children aged 10–12 years, therefore, allows for the identification of early vulnerabilities before disordered eating patterns become more entrenched. This developmental window precedes the broader biological and sociocultural changes typically associated with puberty and adolescence, during which eating-pathology prevalence and gender disparities often intensify [2,4,5]. Examining subclinical symptomatology at this stage provides an opportunity to better understand early eating-related concerns and differentiate them from clinically diagnosed eating disorders that commonly manifest later in development [1]. This developmental focus also allows for the identification of potentially modifiable psychological factors during a formative period, which may inform early preventive efforts.

Eating disorder symptomatology, encompassing both psychological and behavioral aspects of subclinical eating concerns, has been associated with later development of clinical eating disorders [6]. Longitudinal studies have demonstrated that subclinical symptoms in adolescence, such as drive for thinness and body dissatisfaction, tend to stabilize over time, while bulimia and BMI may increase [7]. This progression highlights the importance of identifying early indicators that may be linked to symptom persistence or escalation [8,9]. Early identification may reduce the likelihood of chronicity and mitigate the substantial personal and societal costs associated with long-term intervention programs [10]. This is particularly relevant given evidence that low body esteem can remain stable from approximately age 11 onward, suggesting that critical aspects of body image development may occur before this age and warrant attention during preadolescence [11].

A high prevalence of disordered eating behaviors in young people remains a significant concern due to their association with adverse psychosocial, physical, and mental health outcomes and their potential link to later clinical eating disorders [1]. Once manifested, clinical eating disorders are associated with serious health consequences, including increased mortality and substantial economic burden [1]. The chronic nature of these conditions and their functional impairment further emphasize the importance of preventive efforts during late childhood and early adolescence, when body dissatisfaction and related psychological factors become increasingly salient [4,5].

A growing body of research on disordered eating in Cyprus highlights substantial levels of disturbed attitudes and behaviors beginning in early adolescence and extending into adulthood. National and large-scale school-based studies involving adolescents aged 12–18 consistently report concerning prevalence, with approximately 16% to 35% of girls and 8% to 19% of boys scoring above the recommended diagnostic cutoff on the EAT-26, depending on the sampling year and methodology. These adolescents typically present with heightened appearance dissatisfaction, increased weight-related distress, and greater endorsement of sociocultural ideals surrounding thinness and shape. Longitudinal comparisons further suggest increases in bulimia-related symptoms and maladaptive eating habits over time, particularly among female groups [12].

Findings from young-adult and middle-aged samples further indicate that eating-related impairments may persist beyond adolescence; in some cases, adults demonstrate elevated engagement in problematic weight-control practices such as binge-eating episodes and the use of dieting aids, suggesting developmental continuity rather than remission in certain individuals [13].

Notably, existing studies in Cyprus primarily begin assessing adolescents at or after age 12, by which time body dissatisfaction, dieting efforts, and disordered symptoms are already visible and relatively established. In contrast, the present study focuses on children aged 10–12 years, a developmental period preceding the consolidation of adolescent symptom patterns. Investigating this younger age group allows for examination of early psychological correlates of eating-related symptomatology before later adolescent intensification.

The aim of the present study was to examine early psychological correlates of disordered-eating symptomatology among preadolescent children aged 10–12 years. Specifically, the study investigated the prevalence of clinically elevated disordered-eating risk and examined whether body-figure discrepancy and thinness-related attribution patterns were associated with disordered eating beyond the effects of gender and BMI. The study addressed the following research questions: (1) What is the prevalence of clinically elevated disordered-eating risk (ChEAT-26 ≥ 20), and does it differ by gender? (2) Is body-figure discrepancy (ideal–perceived figure) associated with disordered-eating symptom severity and clinical risk status beyond gender and BMI? (3) Are thinness-related attribution patterns (thin-positive social–emotional bias, thin-negative attribution, and thin body-competence attribution) associated with disordered-eating symptomatology, and do these associations vary by gender?

## 2. Materials and Methods

### 2.1. Participants and Procedure

The National Bioethics Committee for Biomedical and Clinical Research approved this study (Ref. No. ΕΕΒΚ/ΕΠ 2020/56). Participants were recruited using a snowball sampling technique. Data collection was conducted using printed questionnaires due to the sample’s age range. Initially, parents were approached via the school and provided with both verbal and written information regarding the aims of the study. After parents were fully informed, children were also briefed on the study’s purpose and their right to withdraw at any time. Once joint parent–child consent was obtained, the parents signed a written consent form allowing their child to participate. Inclusion criteria included attending 10th–12th grade (10–12-year-olds), being fluent in Greek, and both parents being Greek–Cypriot. Children who were eligible and wished to take part were required to read the information sheet and then proceed to complete the questionnaire package. The information sheet clearly stated the voluntary nature of participation and emphasized that withdrawal was possible at any point without penalty. It also outlined the study’s aims and highlighted issues of anonymity, confidentiality, and data protection. The questionnaire sequence was as follows: demographic information, CBIS scale, and ChEAT. Completion time was approximately 30 min.

The overall sample (see Table 1) consisted of 227 children from Cyprus (M_age_ = 11.22 years, SD = 1.09), of whom 105 (46.3%) were boys, and 122 (53.7%) were girls. Among boys, 31.4% were aged 10, 11.4% were aged 11, and 57.1% were aged 12, while among girls, 18.9%, 18.9%, and 62.3% fell into these respective age categories. Mean self-reported height was 1.55 m (SD = 0.13; range 1.20–1.67), and mean self-reported body weight was 50.80 kg (SD = 15.57; range 23–80). Based on self-reported height and weight, BMI was calculated and indicated a mean BMI of 20.58 (SD = 4.16; range 11.51–34.62). Furthermore, 104 children (45.8%) lived in urban areas and 123 (54.2%) in rural areas.

### 2.2. Measures

#### 2.2.1. Disordered Eating

To assess disordered eating attitudes, the Greek version of the Children’s Eating Attitudes Test–26 (ChEAT-26) was used [14,15]. The ChEAT-26 is a self-report questionnaire designed to assess eating-related attitudes and behaviors associated with disordered eating among children and early adolescents. The instrument consists of 26 items that capture concerns related to dieting, preoccupation with food and body weight, fear of weight gain, and maladaptive eating behaviors. Participants respond to each item on a 6-point Likert-type scale ranging from “never” to “always”. Higher scores indicate greater levels of disordered eating attitudes, with a total score higher than 20 indicating a possible presence of an eating disorder. The measure has demonstrated satisfactory psychometric properties in child and adolescent populations and is appropriate for use with children from approximately 8 years of age. For the current sample, the alpha coefficient was 0.86.

#### 2.2.2. Body Image and Self-Assessment, Size Acceptance, and Shape Satisfaction Questionnaire

Facets of body image were assessed using the Body Image and Self-Assessment, Size Acceptance, and Shape Satisfaction Questionnaire [16]. The questionnaire was developed to assess body image, acceptance of body size, and satisfaction with body shape. In this study, the 18 items focused on the child’s perceptions of their own weight, how they believe their family and friends perceive it, their current level of satisfaction with their weight, and their perceived body type. The questionnaire includes seven child silhouettes representing different body types. Participants are asked to select the silhouette that, in their view, represents their own body type, the one they want to look like, the most attractive child, the most awkward, the strongest and the weakest, the happiest and the saddest, the child who receives the most respect, and the child who is least respected. Based on one’s responses on which silhouette looks like them and which they would like to look like, the body-figure discrepancy was calculated and used as a variable of interest (ideal–perceived figure) [16].

#### 2.2.3. Computation of Thinness-Attribution Indices

Children’s evaluative judgments regarding body size were also assessed through their selections among the seven male/female body silhouettes (1 = thinnest, 7 = largest). For each attribute used, participants indicated which figure best represented a target characteristic. Responses were coded numerically according to the selected figure and subsequently transformed into three theoretically derived composite indices. Thinness-related attribution patterns were operationalized using composite indices developed specifically for the present study. The indices were theoretically grounded in sociocultural and cognitive-developmental models of body image, which posit that children begin forming evaluative associations between body size and socially desirable, emotional, and competence-related attributes prior to adolescence. These evaluative schemas are thought to reflect early internalization of appearance-based norms, including the privileging of thinness and the stigmatization of larger body sizes.

Given the absence of validated child-specific measures capturing these attributional dimensions, composite indices were constructed using silhouette-based evaluative judgments. This visually anchored approach was selected to ensure developmental appropriateness for children aged 10–12 years and to minimize reliance on abstract verbal reasoning. Composite scoring was employed to enhance reliability and reduce item-level variability, with higher scores reflecting stronger thin-preference bias across domains. Internal consistency estimates (Cronbach’s α) for each index are reported below.

- Thin-Positive Social–Emotional Bias Index. This index reflected the tendency to assign socially desirable and emotionally positive characteristics to thinner bodies. Children selected which figure was most “beautiful”, “respected”, and “happy”. Each item was reverse-coded so that higher scores indicated assignment of positive attributes to thinner silhouettes (i.e., 8 − figure selected). The three items were summed to produce a total score ranging from 3 to 21, with higher scores indicating greater thin-positive bias. The Cronbach’s alpha for this index was 0.71.
- Thin-Negative Attribution Index. This index assessed whether negative emotional or social states were attributed to larger rather than thinner bodies. Children selected which figure was “least respected” and “most sad”. Each item was reverse-coded (8 − figure selected) such that higher scores indicated that negative attributes were assigned to larger figures and, correspondingly, positive evaluations were attributed to thinner figures. The two items were summed to yield a total score ranging from 2 to 14. The Cronbach’s alpha for this index was 0.69.
- Thin Body-Competence Attribution Index. This index captured perceptions of physical competence associated with body size. Children selected which figure was “strongest”, “most clumsy”, and “weakest”. All three items were reverse-coded (8 − figure selected) such that higher scores denoted attribution of physical competence and ability to thinner figures. The resulting composite ranged from 3 to 21, with higher values indicating a stronger preference for competence-based thinness. The Cronbach’s alpha for this index was 0.80.

### 2.3. Statistical Analyses

Statistical analyses were performed using SPSS (Version 30; IBM Corp., Armonk, NY, USA). Descriptive statistics (means, standard deviations, frequencies, and percentages) were calculated for demographic and study variables, and normality was assessed. Internal consistency of the ChEAT-26 was assessed using Cronbach’s alpha. Independent-samples *t*-tests were conducted to examine gender differences in body-figure discrepancy. Chi-square tests were used to evaluate gender differences in the proportion of participants scoring at or above the ChEAT-26 clinical cutoff (≥20). Pearson correlation coefficients were computed to assess associations between thinness-attribution indices and disordered eating. Hierarchical multiple regression analyses were performed to examine whether body-figure discrepancy predicted ChEAT-26 total scores beyond gender and BMI. In Step 1, gender and BMI were entered; body-figure discrepancy was entered in Step 2.

Additional linear regression models examined the predictive value of thinness-attribution indices, including models that controlled for gender and BMI and tested gender interaction effects. Binary logistic regression analysis was conducted to determine whether body-figure discrepancy predicted classification above the clinical cutoff (ChEAT-26 ≥ 20). Statistical significance was set at *p* < 0.05 (two-tailed).

## 3. Results

Parental appearance-related characteristics and weight-related feedback are presented in Table 2. Most children reported that their mothers paid considerable attention to their own appearance (73.2%), compared to 55.4% for fathers. Regarding the importance parents placed on the child’s own appearance, most participants perceived this importance as relatively high (36.1% “Quite a lot,” 35.7% “A lot,” and 13.2% “Excessive”). A similar pattern was observed for parental emphasis on diet (38.5% “Quite a lot,” 35.4% “A lot,” and 13.3% “Excessive”). Additionally, 42.7% of children reported having thought of themselves as “fat” at some point. Collectively, these findings indicate that parental appearance attitudes and weight-related communication are prominent in children’s environments during late childhood.

### 3.1. Prevalence of Clinically Elevated Disordered-Eating Risk

The prevalence of clinically elevated disordered-eating risk (ChEAT-26 ≥ 20) in the total sample was 16.3%. Gender-specific rates indicated that 14.5% of boys and 18.2% of girls scored above the threshold. A chi-square test of independence demonstrated that this difference was not statistically significant, *χ*^2^ (1) = 0.39, *p* = 0.532. Although the gender difference was nonsignificant, the overall prevalence indicates that approximately one in six children already present clinically concerning levels of eating-related symptomatology at ages 10–12.

### 3.2. Body-Figure Discrepancy and Disordered-Eating Symptomatology

#### 3.2.1. Gender Differences in Figure–Ideal Discrepancy

An independent-samples *t*-test was conducted to examine whether boys and girls differed in the discrepancy between their perceived and ideal body figures. Results showed that boys reported an average discrepancy score of *M* = 0.52 (SD = 1.66), whereas girls reported a slightly higher mean score of *M* = 0.68 (SD = 1.37). This difference was not statistically significant, *t* (225) = −0.76, *p* = 0.446, indicating that girls did not differ from boys in their desire to change their body figure. The effect size was very small (Cohen’s d = −0.10, 95% CI = −0.36, 0.16), suggesting negligible gender-based variation in figure dissatisfaction at this developmental stage.

#### 3.2.2. Predicting Disordered Eating from Figure–Ideal Discrepancy

A hierarchical multiple regression was conducted to determine whether body-figure discrepancy predicted eating-related symptomatology above and beyond gender and BMI. In Step 1, gender and BMI were entered and did not significantly predict disordered eating, accounting for only 0.9% of the variance (*R*^2^ = 0.009, *F* (2, 223) = 0.97, *p* = 0.379). In Step 2, the figure discrepancy was added to the model and significantly improved prediction, explaining an additional 5.0% of variance (*R*^2^ = 0.050; *F* (1, 222) = 11.80, *p* < 0.001), resulting in a total explained variance of 5.9%. Although statistically significant, the magnitude of explained variance was modest, indicating that body-figure discrepancy accounts for a limited proportion of variability in symptom severity.

In the complete model, figure discrepancy emerged as a significant predictor of ChEAT-26 symptoms, *β* = 0.241, *t* = 3.44, *p* < 0.001, *B* = 1.22 (95% CI [0.52, 1.92]), indicating that for each one-unit increase in discrepancy, ChEAT-26 scores increased by approximately 1.2 points. Gender and BMI were nonsignificant in Step 2 (gender, *β* = 0.037, *p* = 0.572; BMI, *β* = −0.012, *p* = 0.871), suggesting that body-figure discrepancy was associated with symptom severity independent of BMI.

A binary logistic regression was performed to evaluate whether body-figure discrepancy predicted membership in the elevated-risk category (EAT-26 ≥ 20). In the model containing only gender and BMI (Step 1), the predictors did not jointly reach statistical significance *(χ*^2^ (2) = 4.21, *p* = 0.122), with a Nagelkerke *R*^2^ of 0.032. When figure discrepancy was entered (Step 2), there was a significant improvement in model fit, *χ*^2^ (1) = 5.81, *p* = 0.016, and the full model was significant, *χ*^2^ (3) = 10.02, *p* = 0.018, increasing explained variance to 7.4% (Nagelkerke *R*^2^ = 0.074). This proportion of explained variance was small, suggesting that additional factors contribute to clinical risk classification. Most importantly, figure discrepancy significantly predicted risk classification, *Wald* = 5.44, *p* = 0.020. The odds ratio indicated that each additional unit of discrepancy increased the likelihood of being above the clinical threshold by 37%, OR = 1.37, 95% CI [1.05, 1.79]. Gender was nonsignificant (OR = 0.99, *p* = 0.978); BMI did not significantly increase risk (OR = 1.04, *p* = 0.368) once discrepancy was included in the model.

### 3.3. Thinness-Related Attribution Patterns and Disordered-Eating Symptomatology

Pearson correlation coefficients are presented in Table 3. None of the thinness-related attribution indices were significantly associated with ChEAT-26 total scores. Thin-positive bias (*r* = 0.08, *p* = 0.270), thin-negative attribution (*r* = −0.09, *p* = 0.209), and thin body-competence attribution (*r* = 0.00, *p* = 0.970) were unrelated to eating-related symptom severity. However, the attribution indices were significantly intercorrelated. Thin-positive bias was negatively associated with thin-negative attribution (*r* = −0.34, *p* < 0.001) and body-competence attribution (*r* = −0.19, *p* = 0.006). Thin-negative attribution was positively associated with body-competence attribution (*r* = 0.21, *p* = 0.002), suggesting that children’s evaluative patterns toward body size form a coherent attitudinal structure, though not yet linked to symptom expression.

#### 3.3.1. Multiple Regression with All Three Indices

A multiple regression analysis was then conducted with the ChEAT-26 total score as the dependent variable and all three thinness-attribution indices entered simultaneously as predictors. The overall model was not significant, *F* (3, 200) = 0.69, *p* = 0.561, and explained only 1.0% of the variance in disordered eating, *R*^2^ = 0.010, adjusted *R*^2^ = −0.005. None of the predictors emerged as significant: thin-positive bias, *β* = 0.06, *p* = 0.434; thin-negative attribution, *β* = −0.07, *p* = 0.380; and thin body-competence attribution, *β* = 0.01, *p* = 0.895.

#### 3.3.2. Simple Regressions for Each Index

To further clarify these effects, separate simple linear regressions were run for each index. When entered alone, thin-positive bias did not significantly predict ChEAT-26 scores, *F* (1, 208) = 1.22, *p* = 0.270, *R*^2^ = 0.006, *β* = 0.08. Similarly, thin-negative attribution failed to predict symptomatology, *F* (1, 212) = 1.59, *p* = 0.209, *R*^2^ = 0.007, *β* = −0.09, and thin body-competence attribution showed virtually no association with ChEAT-26, *F* (1, 212) = 0.001, *p* = 0.970, *R*^2^ < 0.001, *β* = 0.00.

#### 3.3.3. Hierarchical Regressions Controlling for Gender and BMI

Finally, hierarchical regression models tested whether any of the indices predicted disordered eating beyond the effects of gender and BMI. In each model, gender and BMI were entered at Step 1, followed by one thinness-attribution index at Step 2.

For the thin-positive bias model, Step 1 (gender and BMI) was not significant, R^2^ = 0.004, *F* (2, 207) = 0.38, *p* = 0.687. Adding a thin positive bias at Step 2 did not significantly improve prediction (Δ*R*^2^ = 0.009, Δ*F*(1, 206) = 1.78, *p* = 0.183); the index remained nonsignificant in the final model (*β* = 0.10, *p* = 0.183).

For the thin-negative attribution model, Step 1 again failed to reach significance: *R*^2^ = 0.006, *F*(2, 211) = 0.66, *p* = 0.520. Step 2, adding the negative-attribution index, produced a small, nonsignificant increase in explained variance (Δ*R*^2^ = 0.007; Δ*F*(1, 210) = 1.48; *p* = 0.226); the index was not a unique predictor of symptoms *(β* = −0.08; *p* = 0.226).

Finally, for the body-competence model, Step 1 (gender and BMI) was again nonsignificant, *R*^2^ = 0.006, *F* (2, 211) = 0.69, *p* = 0.504, and adding thin body-competence attribution at Step 2 did not change model fit, Δ*R*^2^ < 0.001, Δ*F* (1, 210) = 0.01, *p* = 0.938; the competence index was not related to disordered eating, *β* = −0.01, *p* = 0.938.

A hierarchical regression model tested whether gender moderated the association between thin-positive bias and eating-related symptoms. Gender and BMI were entered at Step 1 and did not significantly predict ChEAT-26 scores, *R*^2^ = 0.004, *F* (2, 207) = 0.38, *p* = 0.687. Thin-positive bias was entered at Step 2 and did not significantly improve prediction, ΔR^2^ = 0.008, *F* (1, 206) = 1.78, *p* = 0.183. Finally, the interaction term Thin-Positive Bias × Gender was added at Step 3 and did not account for additional variance in eating symptomatology (Δ*R*^2^ < 0.005, ns), indicating that gender did not moderate this association. Together, these results suggest that thin-positive attribution tendencies do not significantly predict eating-related pathology at ages 10–12 and that this association does not differ between boys and girls.

## 4. Discussion

The present study examined psychological correlates of disordered-eating symptomatology among preadolescent children aged 10–12 years, with a specific focus on body-figure discrepancy and thinness-related attribution patterns. By targeting a developmental period that precedes the typical increase in disordered eating during adolescence, this study aimed to clarify whether measurable eating-related concerns and associated psychological variables are already present in late childhood.

### 4.1. Prevalence of Disordered-Eating Risk in Late Childhood

A notable finding was that approximately one in six children (16.3%) scored at or above the clinical cutoff on the ChEAT-26, indicating clinically elevated eating-related risk. This prevalence did not differ significantly between boys and girls, suggesting that sex disparities commonly observed during adolescence are not yet fully expressed in late childhood. These findings align with prior research indicating that gender differences in disordered eating tend to widen during adolescence, particularly following pubertal changes and increasing sociocultural pressures related to appearance and weight [5,8].

The observed prevalence is substantially lower findings from Cypriot adolescent samples aged 12 years and older, where disordered-eating risk has been reported in approximately 20–35% of girls and 8–19% of boys [12], possibly due to the use of a different screening measure. It is, however, almost identical with more recent studies conducted in Cyprus [17,18,19]. Importantly, the presence of clinically meaningful risk prior to adolescence supports longitudinal evidence suggesting that subclinical eating disorder symptoms often emerge early and may stabilize or intensify over time rather than remit spontaneously [6,7]. From a public health perspective, these findings underscore the importance of implementing preventive interventions before adolescence, rather than waiting until gender differences and symptom severity become more pronounced.

These results are also consistent with the literature highlighting that body dissatisfaction and disordered eating symptoms are often well established by early adolescence [2] and that early assessment is critical for detecting emerging pathology before full clinical manifestation [1]. Identifying risk at this young age reinforces the importance of targeting early indicators to prevent escalation to more severe, diagnosable conditions [8,9].

### 4.2. Body-Figure Discrepancy as an Early Psychological Risk Factor

A central contribution of this study is the finding that body-figure discrepancy—the mismatch between perceived and ideal body figures—was a significant predictor of eating-related symptomatology in late childhood. Greater discrepancy was associated with higher ChEAT-26 scores and an increased likelihood of belonging to the clinically elevated risk group, even after controlling for gender and BMI. These findings highlight body-image dissatisfaction as a significant correlate of eating-related symptomatology in late childhood. It should be noted that the proportion of explained variance was modest (approximately 6–7%), indicating that while body-image discrepancy is statistically associated with eating-related concerns, it represents only one of multiple contributing factors.

Indeed, this result supports evidence suggesting that the critical period for body image development often occurs before the age of 11 [11], indicating that body dissatisfaction at this stage may reflect an early psychological marker associated with elevated eating-related symptoms. While previous research has largely focused on adolescents and young adults [20,21,22], the present findings extend this literature by demonstrating that body-image discrepancy predicts disordered eating in a younger cohort, thereby refining developmental models of disordered eating risk.

Notably, the predictive role of body-figure discrepancy was independent of BMI, suggesting that psychological dissatisfaction, rather than objective body size, accounts for meaningful variance in early eating-related symptoms. This aligns with prior findings indicating that children across weight categories may experience comparable vulnerability to disordered eating when body dissatisfaction is present [11]. Furthermore, the emergence of this relationship prior to adolescence extends earlier evidence from adolescent and adult populations [7,9].

### 4.3. Thinness-Related Attribution Patterns: Attitudes Without Symptom Expression

Contrary to expectations, children’s thinness-related attribution patterns—including thin-positive social–emotional bias, thin-negative emotional attributions, and thin body-competence attributions—did not significantly predict eating-related symptomatology, nor were these associations moderated by gender. Although these attribution indices were intercorrelated, indicating a coherent evaluative structure toward body size, they did not translate into measurable differences in disordered eating symptoms at this developmental stage.

This finding is consistent with perspectives suggesting that the internalization of weight bias and its manifestation as a predictor of disordered eating may require a more extended developmental period [23]. While children aged 10–12 appear to hold structured beliefs about body size, these attitudes may not yet be sufficiently internalized or integrated into self-evaluative processes to influence eating-related behaviors [24,25]. Developmental models propose that thin-ideal internalization and appearance-based self-worth intensify during early adolescence, coinciding with increased peer comparison, pubertal changes, and social evaluation [26,27], which may explain the absence of significant effects in the present sample.

### 4.4. Theoretical and Preventive Implications

The present findings emphasize the presence of clinically relevant eating-related risk and the predictive significance of body-figure discrepancy in preadolescent children. Identifying such early markers of vulnerability is essential for proactive intervention efforts aimed at preventing the progression to more severe psychopathology [28]. By identifying body-image discrepancy as a salient correlate of eating-related symptomatology prior to adolescence, this study contributes to a more nuanced understanding of the developmental trajectories of subclinical disordered eating [1].

The lack of association between thinness-related attributions and disordered eating suggests that preventive interventions during late childhood may benefit from including a focus on body dissatisfaction and self-perception rather than targeting thin-ideal beliefs alone. Longitudinal research tracking the co-development of body perception, weight bias internalization, and eating behaviors across childhood and adolescence is needed to clarify when these constructs become behaviorally relevant [23]. Such work would also support the development of child-specific assessment tools that move beyond reliance on BMI and more accurately capture internalized weight bias and body image processes [24,29].

Finally, intervention approaches that emphasize positive psychological processes, such as body appreciation [30], and that foster environments de-emphasizing weight and appearance norms may promote positive body image and reduce the likelihood of later disordered eating [31].

### 4.5. Limitations and Future Research

Several limitations should be considered when interpreting the present findings. First, the cross-sectional design precludes causal inference about the directionality of the association between body-figure discrepancy and disordered eating. Although discrepancy emerged as a significant predictor of eating-related symptomatology, longitudinal research is needed to determine whether body dissatisfaction precedes and contributes to the escalation of symptoms across adolescence or whether reciprocal processes are involved.

Second, the use of self-report measures may introduce shared method variance and potential response biases, particularly in a young sample. Importantly, height and weight were self-reported by the students and subsequently used to calculate BMI. Given the documented tendency for children and adolescents to misreport anthropometric data, BMI estimates may be subject to measurement error, which could have attenuated associations between weight status and eating-related risk. Future studies should incorporate objectively measured height and weight to enhance accuracy.

Third, the sample was recruited using a snowball sampling approach and included only Greek–Cypriot children, which may limit its generalizability to more diverse populations or cultural contexts. Replication in larger, more representative, and cross-cultural samples would strengthen confidence in the developmental generalizability of these findings. Finally, it should be noted that the thinness-attribution indices were newly constructed for the present study and should therefore be considered exploratory; further research is needed to establish their psychometric properties and replicability in independent samples.

Future research would benefit from longitudinal designs tracking children across the transition into adolescence to clarify when thinness-related attribution patterns become behaviorally relevant and whether they interact with pubertal timing, peer influences, or media exposure. Additionally, incorporating multi-informant data (e.g., parent or teacher reports) and experimental or intervention-based methodologies may help identify modifiable mechanisms underlying early body-image discrepancy. Finally, investigating protective factors—such as body appreciation, parental communication styles, and school-based prevention programs—may inform developmentally tailored early intervention strategies aimed at reducing the progression from body dissatisfaction to clinically significant disordered eating.

## 5. Conclusions

The present study examined early psychological correlates of disordered-eating symptomatology among children aged 10–12 years. Approximately one in six participants scored at or above the clinical cutoff on the ChEAT-26, indicating that clinically elevated eating-related concerns are already present in late childhood, prior to the widening gender disparities typically observed during adolescence.

Body-figure discrepancy was significantly associated with both symptom severity and likelihood of exceeding the clinical risk threshold, independent of gender and BMI. Although the proportion of explained variance was modest, these findings suggest that perceived mismatch between ideal and current body figure is associated with higher levels of eating-related symptomatology.

In contrast, thinness-related attribution patterns were internally coherent but not associated with disordered eating in this age group, indicating that such attitudinal structures may not yet translate into symptom expression during late childhood.

Given the cross-sectional nature of the study, conclusions regarding developmental sequencing or causality cannot be drawn. Nevertheless, the presence of clinically relevant symptom levels and the association between body-image discrepancy and eating-related concerns support consideration of early monitoring and prevention efforts before adolescence.

Future longitudinal research is needed to clarify how body dissatisfaction, weight-related attitudes, and eating behaviors co-develop across the transition into adolescence and whether early body-image discrepancy prospectively predicts later clinical outcomes.

## Figures and Tables

**Table 1:** Demographic characteristics of the sample (N = 227).

Variable	Boys (n = 105)	Girls (n = 122)	Total (N = 227)
Age (years)			
10	33 (31.4%)	23 (18.9%)	56 (24.7%)
11	12 (11.4%)	23 (18.9%)	35 (15.4%)
12	60 (57.1%)	76 (62.3%)	136 (59.9%)
Mean Age (SD)			11.22 (1.09)
Height (m)			1.55 (0.13)
Weight (kg)			50.80 (15.57)
BMI			20.58 (4.16)
Residence			
Urban			104 (45.8%)
Rural			123 (54.2%)

**Table 2:** Parental appearance, parental importance on child’s appearance and diet, parental weight status, and feedback received by children (N = 227).

Variable	Response Options	n	% (Valid)
Mother pays great attention to her own appearance	Yes	164	73.2
	No	63	26.8
Father pays great attention to his own appearance	Yes	124	55.4
	No	103	44.6
Parents’ importance placed on the child’s appearance	Not at all	4	1.8
	A little	30	13.2
	Quite a lot	82	36.1
	A lot	81	35.7
	Excessive	30	13.2
Parents’ importance placed on the child’s diet	Not at all	7	3.1
	A little	22	9.7
	Quite a lot	87	38.5
	A lot	80	35.4
	Excessive	31	13.3
Children have thought they are fat	Yes	98	42.7
	No	129	57.3

**Table 3:** Correlational analyses.

Variable	1	2	3	4
1. ChEAT-26	—	0.08	−0.09	0.00
2. Thin-Positive Bias	0.08	—	−0.34 **	−0.19 **
3. Thin-Negative Attribution	−0.09	−0.34 **	—	0.21 **
4. Thin Body Competence	0.00	−0.19 **	0.21 **	—

** = *p* < 0.001.

## Data Availability

Data available upon request to the corresponding author.
